# Hippocampal Atrophy in Systemic Lupus Erythematosus Patients without Major Neuropsychiatric Manifestations

**DOI:** 10.1155/2020/2943848

**Published:** 2020-06-12

**Authors:** Shuang Liu, Yuqi Cheng, Yueyin Zhao, Aiyun Lai, Zhaoping Lv, Zhongqi Xie, Bibhuti Upreti, Xiangyu Wang, Xiufeng Xu, Chunrong Luo, Hongjun Yu, Baoci Shan, Lin Xu, Jian Xu

**Affiliations:** ^1^Department of Rheumatology and Immunology, First Affiliated Hospital of Kunming Medical University, Kunming 650032, China; ^2^Yunnan Key Laboratory of Laboratory Medicine, Kunming 650032, China; ^3^Department of Psychiatry, First Affiliated Hospital of Kunming Medical University, Kunming 650032, China; ^4^Magnetic Resonance Imaging Center, The First Hospital of Kunming, Kunming 650011, China; ^5^Key Laboratory of Nuclear Analysis, Institute of High Energy Physics, Chinese Academy of Sciences, Beijing 100049, China; ^6^Key Laboratory of Animal Models and Human Disease Mechanisms, Kunming Institute of Zoology, Chinese Academy of Sciences, Kunming 650223, China

## Abstract

This study was conducted to explore hippocampal structural changes and their possible associations with clinical characteristics, emotional status, and treatment regimens in patients with systemic lupus erythematosus (SLE) without major neuropsychiatric manifestations (non-NPSLE). Eighty-five non-NPSLE patients with normal conventional magnetic resonance imaging (MRI) and seventy-seven matched healthy control (HC) subjects were recruited. All participants underwent the standard high-resolution volumetric MRI. The bilateral hippocampal volume (HIPV) and hippocampal density (HIPD) were calculated, respectively, for each participant. We found that the bilateral HIPV and HIPD of the SLE patient group were significantly less than those of the HC group. The bilateral HIPV of female patients were significantly less than those of male patients. The SLE disease activity index (SLEDAI) was negatively correlated with the bilateral HIPV and the right HIPD. Urine protein quantity was negatively correlated with the bilateral HIPV and HIPD. Hydroxychloroquine (HCQ) showed a protective effect on right HIPV. In conclusion, we found that the early hippocampal atrophy could occur before obvious neuropsychiatric manifestations and might be associated with SLE disease activity and organ damages. Early detection and intervention of hippocampal damage might prevent the progression to NPSLE. More studies are needed to fully understand the underlying mechanisms of hippocampal atrophy in SLE.

## 1. Introduction

Systemic lupus erythematosus (SLE) is an autoimmune disease with multiorgan involvement. It is characterized by high titers of various serum antibodies targeting nuclear or cytoplasmic antigens. Glucocorticoids (GC) and immunosuppressive agents (ISA) are used to help patients reach the target of remission or low disease activity. Hydroxychloroquine (HCQ), cyclophosphamide (CTX), and Mycophenolate Mofetil (MMF) are widely used ISA (1). The central nervous system (CNS) is commonly involved in SLE (2, 3). Brain atrophy has been detected in SLE patients using several neuroimaging techniques. It is often associated with clinical manifestations in SLE patients and sometimes even in patients without obvious CNS signs and symptoms (4, 5). The hippocampus is located in the temporal lobe of the brain and plays an important role in learning and memory processes. Hippocampal atrophy was found in SLE patients and was related to cognitive dysfunction, disease duration, and history of CNS manifestations (6).

Magnetic resonance imaging (MRI) is one of the most commonly used techniques to evaluate brain abnormalities including brain atrophy. Many patients with mild cognitive impairment have normal conventional MRI findings because conventional MRI is nonspecific or not sensitive enough for delicate structures like hippocampus (4, 6). SLE patients without major neuropsychiatric manifestations are usually considered non-NPSLE patients. In our previous study, we have found that the white matter volume (WMV) of the non-NPSLE patient group was significantly less than that of healthy control (HC) group and that ISA treatment might have a protective effect on WMV (7). Furthermore, in another study, we also found that specific autoantibodies, such as anti-cardiolipin (aCL) antibodies, might contribute to the reduction of grey matter density (GMD) and white matter density (WMD) in non-NPSLE patients. ISA treatment also showed effects in preventing the reduction of GMD and WMD (8). It has been reported that abnormal hippocampal structural changes could be found in non-NPSLE patients with normal conventional brain MRI (9, 10). These results prompted our interest in exploring hippocampal structural changes in non-NPSLE patients by using volumetric MRI.

The underlying mechanisms of hippocampal involvement in SLE patients remain unclear. Various autoantibodies including aCL antibodies and anti-NR2 subtype of the N-methyl-D-aspartate receptor (NMDAR) antibodies were considered to play important roles in the pathogenesis of neuropsychiatric SLE (NPSLE) (8, 11, 12). However, few studies were focused on the relationship between autoantibodies and hippocampal atrophy.

Thus, we conducted this study to explore hippocampal structural changes in non-NPSLE patients and their possible associations with clinical characteristics including specific autoantibodies, disease activity, and emotional status as well as treatment regimens.

## 2. Material and Methods

### 2.1. Subjects

SLE patients were recruited from the inpatient and outpatient facilities of Department of Rheumatology and Immunology of First Affiliated Hospital of Kunming Medical University, a member of the Chinese SLE Treatment and Research Group (CSTAR), from September of 2012 to September of 2014. Each participant went through a standardized protocol and was evaluated by the same investigator throughout the study. All participants had received complete and detailed description of the study and had given written informed consent before enrollment into the study. This research protocol was approved by the Institutional Review Board of Kunming Medical University, Yunnan Province, P. R. China (ClinicalTrials.gov: NCT00703742).

The inclusion criteria included (1) patients diagnosed as SLE according to the 1997 revised American College of Rheumatology (ACR) criteria for the classification of SLE (13), (2) subjects between the ages of 18 and 60, and (3) subjects willing to participate in this study and give written informed consent.

The exclusion criteria included (1) patients fulfilling the ACR diagnostic criteria for rheumatoid arthritis, systemic sclerosis, Sjögren's syndrome (primary or secondary), or other connective tissue diseases and drug-induced SLE; (2) patients with neurological disorders that would affect the brain structure (e.g., history of head trauma, Parkinson's disease, or seizures); (3) patients with major psychiatric manifestations, such as obvious disorganized behaviors and disturbances of consciousness; (4) patients with history of substance abuse; (5) patients who were pregnant or suspected to be pregnant; (6) patients with contraindications to MRI, such as claustrophobia or having cardiac pacemakers; (7) patients with serious clinical conditions that could cause cerebral atrophy, such as history of hypertension, diabetes mellitus, and renal insufficiency; and (8) patients with brain structural abnormalities identified by conventional T1 and T2 weighted MRI.

All 85 SLE patients recruited had undergone full sets of laboratory tests and MRI scans and filled up general questionnaires. A rheumatologist and a neurologist performed complete physical examination including neurological examination on all SLE patients and 77 HC subjects to exclude major disorders. Additionally, psychiatric symptoms were screened by a psychiatrist using the Structured Clinical Interview for Diagnostic and Statistical Manual of Mental Disorders-IV Non-Patient Version (SCID-NP). All participants were Chinese Han people and were right-handed.

### 2.2. Clinical Characteristics of SLE Patients

We recorded the gender, age, and disease duration for each patient. The disease duration was defined as the period from the appearance of initial signs and symptoms of SLE to the day of MRI acquisition. All clinical manifestations and laboratory test results were systematically recorded. Disease activity was measured using SLE disease activity index (SLEDAI). Disease was considered to be active when the SLEDAI score was higher than nine (14, 15). All participants were right-handed as assessed by the Edinburgh Handedness Inventory (16). All clinical data were collected on the day of MRI scans.

The cumulative dosages of GC and ISA were documented through careful interviews and calculated by adding up all the daily dosages. The dosages of oral and intravenous GC were all converted to equivalent dosages of prednisone.

### 2.3. Autoantibodies

Each patient was tested for a set of antibodies, including antinuclear antibody (ANA), anti-double-stranded deoxyribonucleic acid (dsDNA), anti-SSA/Ro 52 kD, anti-SSA/Ro 60 kD, anti-SSB/La, anti-Sm, anti-histone, anti-ribosomal P0, anti-nucleosome, anti-U1 ribonucleoprotein (RNP), and aCL antibodies. ANA was tested with indirect immunofluorescence using Hep-2 cells as substrates (SCIMEDX Corporation, New Jersey, USA); ANA spectrum antibodies were tested with the line immunoassay method using IMTEC kit (IMTEC, Berlin, Germany); aCL antibodies were tested with conventional ELISA (Aesku Diagnostics, Wendelsheim, Germany). All blood samples were drawn on the day of MRI acquisitions.

### 2.4. Image Acquisition

Image acquisitions were performed by an experienced neuroradiologist. MRI sequences were performed on all subjects using a 1.5 T clinical MRI scanner equipped with a birdcage head coil. It was manufactured by General Electric (GE) Company (Twin speed, Milwaukee, WI, USA). Supportive foam pads were used to minimize head movement. A rapid sagittal localizer scan was acquired to ensure alignment. Conventional T1 and T2 MRI scans were taken to exclude obvious structural abnormalities. A set of three-dimensional volumetric structural MRI scans was performed on each subject using a fast spoiled gradient echo sequence (FSPGR) with the following parameters: repetition time (TR)/echo time (TE) = 10.5/2 ms, matrix size = 256 × 256, thickness = 1.8 mm with no interslice gap, field of view = 240 mm, flip angle = 15°, and resolution = 0.94 × 0.94 × 0.9 mm^3^. A total of 172 continuous slices of whole brain data were acquired in axial planes parallel to the anterior commissure-posterior commissure line.

### 2.5. Data Preprocessing and VBM Statistical Analysis

Digital Imaging and Communications in Medicine (DICOM) image data were processed with MRIcro software (version 1.40, Chris Rorden's Neuropsychology Laboratory, University of South Carolina, Columbia, SC, USA; http://www.mricro.com). All data were analyzed by using statistical parametric mapping (SPM) 5 (Wellcome Department of Cognitive Neurology, Institute of Neurology, London, UK; http://www.fil.ion.ucl.ac.uk/) and voxel-based morphometry (VBM) 5 (Department of Neurology, Department of Psychiatry, Friedrich Schiller University Jena, Thuringia, Germany; http://dbm.neuro.uni-jena.de/vbm/vbm5-for-spm5/) software based on Matlab 7.1 (The MathWorks, Inc., Natick, MA, USA). Each individual image was normalized and transformed into the standardized Montreal Neurological Institute (MNI) template, then resampled in 2 × 2 × 2 mm scale. Normalized images were then segmented into grey matter (GM), white matter (WM), and cerebrospinal fluid (CSF). Modulated GM and WM images were separately smoothened to remove noise using a filter with full width at half maximum (FWHM) of 8 mm.

### 2.6. Analysis of Hippocampal Volume (HIPV) and Hippocampal Density (HIPD)

At first, we used the standard GM and WM templates implanted in SPM 5 as the whole brain GM and WM masks to get the total brain volume. Then, we used WFU PickAtlas software (NeuroImaging Tools and Resources Collaboratory, Radiology Informatics and Imaging Laboratory, Department of Radiology, Wake Forest University Health Sciences Medical Center, NC, USA; http://www.nitrc.org/projects/wfu_pickatlas) to get the hippocampal mask. Finally, we retrieved HIPV and HIPD data. Statistical analysis was conducted with SPSS 20.0 (IBM Inc., Armonk, NY, USA). Covariance analysis was performed to analyze the differences in HIPV/HIPD between SLE and HC groups as well as different subgroups of SLE patients. Age and total brain volume were covariates. We used the Bonferroni method for HIPV/HIPD correction in multiple comparisons. Partial correlation analysis, controlling for age and total brain volume, was used to explore possible correlations between disease characteristics and HIPV/HIPD. The results were believed to be statistically significant when *p* < 0.05. All statistical tests were two-sided.

## 3. Results

### 3.1. Demographic Data in SLE and HC Groups

In this study, 85 SLE and 77 HC subjects were recruited. The mean age of SLE patients was 29.28 years (standard deviation (SD) = 7.02, range 18-48) and the mean age of HC subjects was 30.81 years (SD = 7.44, range 18-50). There were no significant differences in age or gender between these two groups. The disease duration in SLE patients ranged from 0.25 to 204 months (mean = 19.90 months, SD = 28.90). The mean SLEDAI was 9.53 (SD = 6.13). Detailed data are showed in Table 1.

Among the 85 SLE patients, 40 (47.1%) patients were positive for anti-Sm antibody, 55 (64.7%) were positive for anti-dsDNA antibody, 45 (52.9%) were positive for anti-SSA/Ro 52 kD antibody, 56 (65.9%) were positive for anti-SSA/Ro 60 kD antibody, 30 (35.3%) were positive for anti-SSB/La antibody, and 10 (11.8%) were positive for aCL antibody.

### 3.2. HIPV/HIPD Differences between SLE and HC Groups

**Figure 1:**
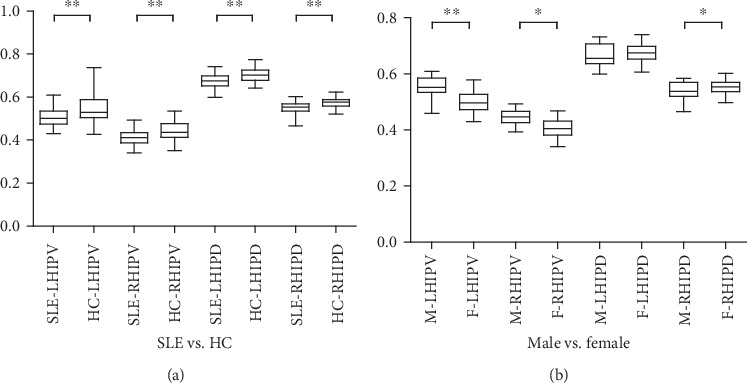
Comparisons of hippocampal volume and density in different groups. (a). Comparisons of hippocampal volume and density between SLE patients and HC group; (b). Comparisons of hippocampal volume and density between male and female SLE patients. ^∗^*p* < 0.05, ^∗∗^*p* < 0.01.

The bilateral HIPV and HIPD of SLE group were significantly less than those of the HC group (see Figure 1(a), Table 2). The bilateral HIPV of male patients were significantly greater than those of female patients. However, the right HIPD of male patients was significantly less than that of female patients, while the left HIPD was comparable between different genders (see Figure 1(b), Table 3).

Due to the gender differences, we reanalyzed the bilateral HIPV and right HIPD in the SLE and HC groups while controlling for gender as a covariate. The adjusted *p* values are shown in Table 2.

### 3.3. Associations between HIPV/HIPD and Clinical Characteristics

All the statistical analyses involving the bilateral HIPV and the right HIPD were done while controlling for gender. We divided patients into two groups: a positive aCL antibody group and a negative aCL antibody group. Bilateral HIPV/HIPD in these two groups showed no significant differences. Similarly, we divided the patients into two groups according to the results of the rest of the antibodies tested and we found that each antibody positivity/negativity pair had no significant differences in their HIPV/HIPD. Patients with active disease (SLEDAI > 9) showed less right HIPD than those with low disease activity or in remission (0.5444 ± 0.0256 vs. 0.5544 ± 0.0214; *p* = 0.032). SLEDAI scores showed negative correlations with bilateral HIPV and right HIPD (*r* = −0.239, *p* = 0.031; *r* = −0.245, *p* = 0.027; *r* = −0.221, *p* = 0.046, respectively). The urine protein quantities were negatively correlated with bilateral HIPV and HIPD (*r* = −0.300, *p* = 0.006; *r* = −0.255, *p* = 0.021; *r* = −0.272, *p* = 0.011; *r* = −0.247, *p* = 0.025, respectively). The total HCQ dose showed a positive correlation with right HIPV (*r* = 0.254, *p* = 0.021). There were no significant correlations between HIPV/HIPD and other factors including disease duration, total GC, total CTX, HAMD, and HAMA scores. Detailed data are showed in Table 4.

## 4. Discussion

In this study, we found a significant reduction in the hippocampal volume and density in non-NPSLE patients when compared to those of the HC group. Female patients showed greater reductions in the hippocampal volume. Hippocampal atrophy was associated with disease activity and urine protein. HCQ showed a protective effect on hippocampal volume.

Although MRI is considered to be a good method to evaluate CNS damage in SLE, conventional MRI findings are often nonspecific or unable to detect any changes in patients with or without NPSLE (17). Hippocampus is crucial in learning and memory processes and plays a role in the regulation of emotional status (18). A temporal progression of hippocampal volume reduction has been reported in SLE patients with cognitive dysfunction (6). Patients in our study had no major neuropsychiatric manifestations or abnormal conventional MRI findings; i.e., they were non-NPSLE patients. Therefore, the hippocampal atrophy implied that brain damage could occur even before the appearance of obvious neuropsychiatric signs and symptoms. Previous studies have also shown that abnormal structural changes, such as hippocampal atrophy, could occur in early phases and affect cognitive function in SLE patients (9, 19, 20). Our study used a quantitative volumetric MRI technique to measure the hippocampal volume and found hippocampal atrophy in non-NPSLE patients.

Gender differences in hippocampal density and volume were found in our study. Bilateral HIPV of female patients were significantly less than those of male patients. It was consistent with another study of our team, in which we found that female SLE patients had significantly lower whole brain grey matter volume (GMV) and WMV than male patients (21). We hypothesized that these findings might be related to the higher estrogen levels in females. Estrogen was considered to be one cause of the higher prevalence of SLE in females. Some researchers believed that gender differences could be found in almost all brain activities and that estrogen could regulate the hippocampus associated learning and memory processes differently in different genders (22). However, when compared with male patients, we also found a greater right HIPD in female patients. When processed by the VBM method, the volume data were modulated and might be more reliable than density data. The higher right HIPD values in females with a *p* value of 0.047 might not be as meaningful as the HIPV differences. Another possible explanation for the variation could be a different aspect of gender differences, the compensation mechanism in females, which has also been mentioned previously in a study (23). More studies are needed to confirm the gender differences and reveal the underlying mechanisms.

The exact roles of autoantibodies in the pathogenesis of brain damage in SLE remain unclear. However, several studies suggest that because blood brain barrier (BBB) lesions could bring high titer auto antibodies in contact with myeloid or glial cells and cause their activation; they could induce production of local cytokines, such as IL-6, which was found elevated in CSF of SLE patients (24–29). Also, some researchers reported that autoantibodies could affect certain brain regions due to BBB lesions and cause organ damages through different pathways (24). However, we failed to find the associations between autoantibodies and hippocampal atrophy. Further investigations are needed to know whether autoantibodies play any role in the pathogenesis of hippocampal atrophy.

On the other hand, we found negative correlations between the HIPV/HIPD and urine protein quantities as well as SLEDAI scores. It implied that early hippocampal atrophy might be associated with SLE disease activity and other organ damages. The associations between early brain atrophy and SLE disease activity were reported by our team earlier (7, 8). Appenzeller et al. reported that SLE patients with white matter lesions had hippocampal atrophy more frequently than patients without it. Furthermore, the hippocampal loss in MRI might become progressive over time in patients with long disease duration (6).

Additionally, we found that HCQ might prevent HIPV reduction. This is consistent with our previous studies which showed that ISA had protective effects on brain atrophy in SLE patients (7, 8, 21). In our studies, ISA included CTX and HCQ. CTX is the most common and efficacious immunosuppressant used for the treatment of lupus. Leung et al. reported that CTX could improve brain demyelinating lesions in SLE (30). Ballok et al. reported that the hippocampus of a NPSLE patient showed decreased neuronal density and suggested that autoimmunity might contribute to the hippocampal damage. They then observed that hippocampal damage could be improved by CTX treatment in Murphy Roths Large-lymphoproliferation (MRL-lpr) mice (31). As an antimalarial drug, HCQ shows good immunomodulation. It could reduce damage accrual, improve survival rate, prevent thrombotic events, improve dyslipidemia, and prevent SLE flares (32, 33). González et al. reported that HCQ was associated with a longer time to neuropsychiatric damage occurrence, which might reveal the protective effect on early brain damage in SLE (34). However, no other study has shown a similar protective effect of HCQ. Also, the exact mechanism of this effect remains unclear. More studies, especially prospective studies and animal experiments to explore it further are warranted.

## 5. Limitations

There are still some flaws in our study. One is that we only used the VBM method to calculate the hippocampal volume even though there were other available methods such as manual tracing and FreeSurfer segmentation. Grimm et al. reported that VBM and FreeSurfer were comparable in calculating the hippocampal volume (35). Wenger et al. reported that FreeSurfer was more reliable in assessing the hippocampal volume in young adults than older ones (36). In our study, we considered the VBM method to be feasible to calculate the hippocampal volume.

Our study shows that hippocampal atrophy detected by volumetric MRI could occur before obvious neurological manifestations in SLE and is one of the primary deficits in SLE. It might be associated with SLE disease activity and other organ damages, especially proteinuria. HCQ might have a protective effect. Thus, early detection and intervention of hippocampal damage might prevent the progression to NPSLE. Further studies are still required to explore the underlying mechanisms of hippocampal involvement in SLE.

## Figures and Tables

**Table 1:** Demographic and clinical characteristics of SLE and HC groups.

	SLE (*n* = 85)	HC (*n* = 77)	*t*	*p* value
Age (year, mean ± SD)	29.28 ± 7.02	30.81 ± 7.44	-1.340	0.182
Female/male	71/14	57/20	2.200 (*χ*^2^)	0.138
Disease duration (m, mean ± SD)	19.90 ± 28.90	NA		
SLEDAI (mean ± SD)	9.53 ± 6.13	NA		
Total GC (g, mean ± SD)	9.46 ± 13.49 (*n* = 67)	NA		
Total CTX (g, mean ± SD)	4.15 ± 3.41 (*n* = 22)	NA		
Total HCQ (g, mean ± SD)	55.06 ± 95.42 (*n* = 38)	NA		

SLE: systemic lupus erythematosus; HC: healthy control; SD: standard deviation; NA: not applicable; SLEDAI: SLE disease activity index; GC: glucocorticoid; CTX: cyclophosphamide; HCQ: hydroxychloroquine.

**Table 2:** Hippocampal volume and density in SLE and HC groups.

	SLE (n = 85)		HC (*n* = 77)		*p* value	Adjusted *p* value
	Mean	SD	Mean	SD		
LHIPV	0.5079	0.0405	0.5463	0.0555	0.001^∗∗^	0.001^∗∗^
RHIPV	0.4132	0.0320	0.4427	0.0406	0.001^∗∗^	0.001^∗∗^
LHIPD	0.6756	0.0305	0.7039	0.0297	<0.001^∗∗^	—
RHIPD	0.5500	0.0237	0.5730	0.0229	<0.001^∗∗^	<0.001^∗∗^

SLE: systemic lupus erythematosus; HC: healthy control; SD: standard deviation; L: left; HIPV: hippocampal volume; R: right; HIPD: hippocampal density. ^∗^*p* < 0.05, ^∗∗^*p* < 0.01.

**Table 3:** Gender differences of hippocampal volume and density in SLE patients.

	Male (*n* = 14)		Female (*n* = 71)		*p* value
	Mean	SD	Mean	SD	
LHIPV	0.5515	0.0399	0.4993	0.0349	0.005^∗∗^
RHIPV	0.4458	0.0284	0.4068	0.0287	0.021^∗^
LHIPD	0.6657	0.0422	0.6775	0.0284	0.118
RHIPD	0.5379	0.0322	0.5524	0.0211	0.047^∗^

SLE: systemic lupus erythematosus; SD: standard deviation; L: left; HIPV: hippocampal volume; R: right; HIPD: hippocampal density. ^∗^*p* < 0.05, ^∗∗^*p* < 0.01.

**Table 4:** Correlations between HIPV/HIPD and clinical characteristics.

	LHIPV		RHIPV		LHIPD		RHIPD
	*r*	*p*	*r*	*p*	*r*	*p*	*r*	*p*
Disease duration (m)	-0.193	0.082	-0.115	0.302	-0.141	0.192	-0.136	0.222
Urine protein (g/d)	-0.300	0.006^∗∗^	-0.255	0.021^∗^	-0.272	0.011^∗^	-0.247	0.025^∗^
SLEDAI	-0.239	0.031^∗^	-0.245	0.027^∗^	-0.183	0.090	-0.221	0.046^∗^
Total GC (g)	-0.215	0.053	-0.127	0.256	-0.204	0.058	-0.148	0.183
Total HCQ (g)	0.111	0.320	0.254	0.021^∗^	0.024	0.825	0.069	0.537
Total CTX (g)	-0.047	0.674	-0.083	0.457	-0.052	0.635	-0.035	0.755
HAMD	0.004	0.969	0.010	0.929	-0.028	0.799	-0.005	0.965
HAMA	-0.022	0.844	0.056	0.616	-0.035	0.745	0.045	0.688

L: left; HIPV: hippocampal volume; R: right; HIPD: hippocampal density; SLICC: Systemic Lupus International Collaborating Clinics/American College of Rheumatology Damage Index for Systemic Lupus Erythematosus; SLEDAI: systemic lupus erythematosus disease activity index; GC: glucocorticoid; HCQ: hydroxychloroquine; CTX: cyclophosphamide. ^∗^*p* < 0.05, ∗∗*p* < 0.01.

## Data Availability

All datasets for this study can be found in the Figshare. Please see the 10.6084/m9.figshare.12017730.v3 for details.

## References

[1] van Vollenhoven R. F., Mosca M., Bertsias G. (2014). Treat-to-target in systemic lupus erythematosus: recommendations from an international task force. *Annals of the Rheumatic Diseases*.

[2] Adelman D. C., Saltiel E., Klinenberg J. R. (1986). The neuropsychiatric manifestations of systemic lupus erythematosus: an overview. *Seminars in Arthritis and Rheumatism*.

[3] Sanna G., Piga M., Terryberry J. W. (2016). Central nervous system involvement in systemic lupus erythematosus: cerebral imaging and serological profile in patients with and without overt neuropsychiatric manifestations. *Lupus*.

[4] Huizinga T. W. J., Steens S. C. A., van Buchem M. A. (2001). Imaging modalities in central nervous system systemic lupus erythematosus. *Current Opinion in Rheumatology*.

[5] Appenzeller S., Pike G. B., Clarke A. E. (2008). Magnetic resonance imaging in the evaluation of central nervous system manifestations in systemic lupus erythematosus. *Clinical Reviews in Allergy and Immunology*.

[6] Appenzeller S., Carnevalle A. D., Li L. M., Costallat L. T. L., Cendes F. (2006). Hippocampal atrophy in systemic lupus erythematosus. *Annals of the Rheumatic Diseases*.

[7] Xu J., Cheng Y., Chai P. (2010). White-matter volume reduction and the protective effect of immunosuppressive therapy in systemic lupus erythematosus patients with normal appearance by conventional magnetic resonance imaging. *The Journal of Rheumatology*.

[8] Xu J., Cheng Y., Lai A. (2015). Autoantibodies affect brain density reduction in nonneuropsychiatric systemic lupus erythematosus patients. *Journal of Immunology Research*.

[9] Kozora E., Brown M. S., Filley C. M. (2010). Memory impairment associated with neurometabolic abnormalities of the hippocampus in patients with non-neuropsychiatric systemic lupus erythematosus. *Lupus*.

[10] Shapira-Lichter I., Vakil E., Litinsky I. (2013). Learning and memory-related brain activity dynamics are altered in systemic lupus erythematosus: a functional magnetic resonance imaging study. *Lupus*.

[11] Lauvsnes M. B., Beyer M. K., Kvaløy J. T. (2014). Association of hippocampal atrophy with cerebrospinal fluid antibodies against the NR2 subtype of the N-methyl-D-aspartate receptor in patients with systemic lupus erythematosus and patients with primary Sjögren's syndrome. *Arthritis & Rhematology*.

[12] Zhang J., Jacobi A. M., Wang T., Berlin R., Volpe B. T., Diamond B. (2009). Polyreactive autoantibodies in systemic lupus erythematosus have pathogenic potential. *Journal of Autoimmunity*.

[13] Hochberg M. C. (1997). Updating the American College of Rheumatology revised criteria for the classification of systemic lupus erythematosus. *Arthritis and Rheumatism*.

[14] Bombardier C., Gladman D. D., Urowitz M. B. (1992). Derivation of the SLEDAI. A disease activity index for lupus patients. *Arthritis and Rheumatism*.

[15] Gladman D. D., Ibañez D., Urowitz M. B. (2002). Systemic lupus erythematosus disease activity index 2000. *The Journal of Rheumatology*.

[16] Oldfield R. C. (1971). The assessment and analysis of handedness: the Edinburgh inventory. *Neuropsychologia*.

[17] Brey R. L. (2007). Neuropsychiatric lupus: clinical and imaging aspects. *Bulletin of the NYU Hospital for Joint Diseases*.

[18] Bartsch T., Wulff P. (2015). The hippocampus in aging and disease: from plasticity to vulnerability. *Neuroscience*.

[19] Gao H. X., Campbell S. R., Cui M. H. (2009). Depression is an early disease manifestation in lupus-prone MRL/lpr mice. *Journal of Neuroimmunology*.

[20] Kozora E., Arciniegas D. B., Filley C. M. (2005). Cognition, MRS neurometabolites, and MRI volumetrics in non-neuropsychiatric systemic lupus erythematosus: preliminary data. *Cognitive and Behavioral Neurology*.

[21] Liu S., Cheng Y., Zhao Y. (2018). Clinical factors associated with brain volume reduction in systemic lupus erythematosus patients without major neuropsychiatric manifestations. *Frontiers in Psychiatry*.

[22] Gillies G. E., McArthur S. (2010). Estrogen actions in the brain and the basis for differential action in men and women: a case for sex-specific medicines. *Pharmacological Reviews*.

[23] Ren T., Ho R. C. M., Mak A. (2012). Dysfunctional cortico-basal ganglia-thalamic circuit and altered hippocampal-amygdala activity on cognitive set-shifting in non-neuropsychiatric systemic lupus erythematosus. *Arthritis and Rheumatism*.

[24] Diamond B., Volpe B. T. (2012). A model for lupus brain disease. *Immunological Reviews*.

[25] Abbott N. J., Mendonça L. L. F., Dolman D. E. M. (2016). The blood-brain barrier in systemic lupus erythematosus. *Lupus*.

[26] Kowal C., DeGiorgio L. A., Nakaoka T. (2004). Cognition and Immunity: Antibody Impairs Memory. *Immunity*.

[27] Trysberg E., Carlsten H., Tarkowski A. (2016). Intrathecal cytokines in systemic lupus erythematosus with central nervous system involvement. *Lupus*.

[28] Baraczka K., Nékám K., Pozsonyi T., Szüts I., Ormos G. (2004). Investigation of cytokine (tumor necrosis factor-alpha, interleukin-6, interleukin-10) concentrations in the cerebrospinal fluid of female patients with multiple sclerosis and systemic lupus erythematosus. *European Journal of Neurology*.

[29] Bravo-Zehnder M., Toledo E. M., Segovia-Miranda F. (2015). Anti-ribosomal P protein autoantibodies from patients with neuropsychiatric lupus impair memory in mice. *Arthritis & Rhematology*.

[30] Leung F. K., Fortin P. R. (2003). Intravenous cyclophosphamide and high dose corticosteroids improve MRI lesions in demyelinating syndrome in systemic lupus erythematosus. *The Journal of Rheumatology*.

[31] Ballok D. A., Woulfe J., Sur M., Cyr M., Sakic B. (2004). Hippocampal damage in mouse and human forms of systemic autoimmune disease. *Hippocampus*.

[32] Ruiz-Irastorza G., Ramos-Casals M., Brito-Zeron P., Khamashta M. A. (2009). Clinical efficacy and side effects of antimalarials in systemic lupus erythematosus: a systematic review. *Annals of the Rheumatic Diseases*.

[33] Costedoat-Chalumeau N., Dunogué B., Morel N., le Guern V., Guettrot-Imbert G. (2014). Hydroxychloroquine: a multifaceted treatment in lupus. *Presse Médicale*.

[34] González L. A., Pons-Estel G. J., Zhang J. (2009). Time to neuropsychiatric damage occurrence in LUMINA (LXVI): a multi-ethnic lupus cohort. *Lupus*.

[35] Grimm O., Pohlack S., Cacciaglia R. (2015). Amygdalar and hippocampal volume: a comparison between manual segmentation, Freesurfer and VBM. *Journal of Neuroscience Methods*.

[36] Wenger E., Mårtensson J., Noack H. (2014). Comparing manual and automatic segmentation of hippocampal volumes: reliability and validity issues in younger and older brains. *Human Brain Mapping*.

